# Retraction: Impact of low-carbon economic policies on the corporate environmental responsibility model in China

**DOI:** 10.1371/journal.pone.0331524

**Published:** 2025-09-04

**Authors:** The PLOS One Editors

The *PLOS One* Editors retract this article [1] because it was identified as one of a series of submissions for which we have concerns about potential manipulation of the publication process, peer review integrity, and authorship. These concerns call into question the validity and provenance of the reported results. We regret that the issues were not identified prior to the article’s publication.

HL agreed with the retraction. HI responded but expressed neither agreement nor disagreement with the editorial decision. MS either did not respond directly or could not be reached.
